# ChatGPT-4.0 vs. Google: Which Provides More Academic Answers to Patients' Questions on Arthroscopic Meniscus Repair?

**DOI:** 10.7759/cureus.76380

**Published:** 2024-12-25

**Authors:** Atahan Eryilmaz, Mahmud Aydin, Cihangir Turemis, Serkan Surucu

**Affiliations:** 1 Orthopedic Surgery, Haseki Training and Research Hospital, Istanbul, TUR; 2 Orthopedic Surgery, Sisli Memorial Hospital, Istanbul, TUR; 3 Orthopedic Surgery, Cesme Alper Cizgenakat State Hospital, Izmir, TUR; 4 Orthopedics and Rehabilitation, Yale University, New Haven, USA

**Keywords:** arthroscopy, artificial intelligence, chatgpt, google, meniscus, surgery

## Abstract

Purpose

The purpose of this study was to evaluate the ability of a Chat Generative Pre-trained Transformer (ChatGPT) to provide academic answers to frequently asked questions using a comparison with Google web search FAQs and answers. This study attempted to determine what patients ask on Google and ChatGPT and whether ChatGPT and Google provide factual information for patients about arthroscopic meniscus repair.

Method

A cleanly installed Google Chrome browser and ChatGPT were used to ensure no individual cookies, browsing history, other side data, or sponsored sites. The term "arthroscopic meniscus repair" was entered into the Google Chrome browser and ChatGPT. The first 15 frequently asked questions (FAQs), answers, and sources of answers to FAQs were identified from both ChatGPT and Google search engines.

Results

Timeline of recovery (20%) and technical details (20%) were the most commonly asked question categories of a total of 30 questions. Technical details and timeline of recovery questions were more commonly asked on ChatGPT compared to Google (technical detail: 33.3% vs. 6.6%, p=0.168; timeline of recovery: 26.6% vs. 13.3%, p=0.651). Answers to questions were more commonly from academic websites in website categories in ChatGPT compared to Google (93.3% vs. 20%, p=0.0001). The most common answers to frequently asked questions were academic (20%) and commercial (20%) in Google.

Conclusion

Compared to Google, ChatGPT provided significantly fewer references to commercial content and offered responses that were more aligned with academic sources. ChatGPT may be a valuable adjunct in patient education when used under physician supervision, ensuring information aligns with evidence-based practices.

## Introduction

Meniscal procedures are the most commonly treated knee injuries, with an incidence between 60 and 70 per 100,000 in the United States [1]. One of the reasons for its high incidence is that surgery is the main treatment for meniscal tears, although conservative treatment may be preferred for some patients [2]. Advantages such as a lower risk of infection, minimal invasiveness, faster recovery, and low complication rates are some other reasons for its popularity [2]. On the other hand, bleeding, infection, blood clots, and nerve or blood vessel damage are some of the potential complications [3,4].

For the last decade, artificial intelligence (AI), which analyzes patterns and extracts relevant data by continuously learning new algorithms, has been used to improve search outcomes, which revolutionized eligibility for online information in almost every industry, from finance to healthcare [5,6]. In 1998, Google, traditionally the most popular search engine in the world, was brought into our lives along with its technologically advancing versions (such as AI), which provided an easily accessible environment for health information with questionable reliability [7]. In 2018, the Chat Generative Pre-trained Transformer (ChatGPT) model by open AI was introduced and has progressed to its latest version, ChatGPT 4.0. This provides a better understanding of complex conversations and more accurate informative responses that result in game-changing effects in orthopedics. Some of these innovative uses of AI are the ability to detect fractures, address meniscus tears, and also answer patients' questions [8-10].

Patients have access to a variety of sources when seeking information about their condition, including their healthcare providers, medical journals, health applications, support groups, and online sources [11]. Various studies show that many patients use the internet to get information about their condition before and after visiting doctors' offices in various branches of medicine, including orthopedics [11,12]. From the physician's standpoint, the physician and the patient are both more satisfied when the physician is aware of the information that patients are attempting to acquire and is able to provide pertinent information in response to their inquiries. This is the reason for an increase in research on the questions that patients pose on the internet [10,13,14].

The purpose of this study was to evaluate ChatGPT and Google for providing reliable and objective information to patients about their questions about arthroscopic meniscus repair. The hypothesis was that ChatGPT would provide more academic frequently asked questions (FAQs) and answers about arthroscopic meniscus repair than Google.

## Materials and methods

Study design

As this study did not examine human subjects, it was exempt from institutional review board approval. This study is a comprehensive review of ChatGPT responses to Google searches for arthroscopic meniscus repair. To minimize the effects of individualized search algorithms, a clean-installed Google Chrome browser (version 127.0.6533.26, Menlo Park, CA) search engine (www.google.com) was used on July 14, 2024. Clean-installed Google Chrome was used to ensure that there were no individual cookies, browsing history, other side data, or sponsored sites. The search was performed on the term "arthroscopic meniscus repair". This term was individually entered into the Google browser, and the first 15 frequently asked questions (FAQs) and answers to FAQs were identified from the "people also ask" section of the Google search engine. The inclusion criteria included search terms "arthroscopic meniscus repair", "meniscus repair surgery", or "meniscus surgery" in the question or the answer section. These questions, answers, and website sources were extracted. The exclusion criteria were duplicated questions and questions that were not relevant to arthroscopic meniscus repair. ChatGPT, a freely available chatbot of OpenAI, was used to extract the most frequently asked questions of patients about arthroscopic meniscus repair.

To avoid the effects of individualized search algorithms, a clean ChatGPT 4.0 account was used to input statements to ChatGPT. The statement "perform a search with the search term "arthroscopic meniscus repair" and record the 15 most frequent questions related to the search term with answers to questions and the website source of answers" was entered into ChatGPT. The top 15 questions, answers, and sources provided by ChatGPT were recorded. In total, 30 questions were recorded (see Table 1).

**Table 1:** Comparison of Google and ChatGPT questions, answers and sources

Google: arthroscopic meniscus repair (question, answer, source)	ChatGPT: arthroscopic meniscus repair (question, answer, source)
Timeline of recovery: What is the recovery time for arthroscopic meniscus repair? Meniscus repair recovery time is typically six to nine months depending on the activities of the patient. Most meniscus repair surgeries require two to four weeks on crutches with a knee brace for the first six weeks. Physical therapy starts right away to help the knee regain range of motion. (Source: academic)	Technical details: What is arthroscopic meniscus repair? Arthroscopic meniscus repair involves stitching torn meniscus tissue to allow it to heal. (Source: academic)
Pain: How painful is arthroscopic meniscus surgery? Arthroscopic meniscus repair is moderately painful. Because more soft tissue surgery is performed, it is more painful than a standard arthroscopy, but less painful than a ligament reconstruction or another procedure that requires drilling holes through the bone. (Source: academic) ​​​​​​	Indications/management: Who needs meniscus repair surgery? Candidates are typically younger individuals with tears that have a good blood supply. (Source: academic)
Risks and complications: What are the symptoms of a failed meniscus repair? Noyes et al. defined the 'failure of the repair' as the persistence of symptoms (swelling, locking, or joint pain) and/or the requirement for repeat knee arthroscopy and meniscectomy. Some studies have found that on repeat arthroscopy, the menisci can be partially healed in the absence of clinical symptoms. (Source: government)	Technical details: What are the different types of meniscus surgeries? Common types include meniscus repair, partial meniscectomy, and meniscus transplant. (Source: academic)
Technical details: Is meniscus repair a major surgery? The surgery requires a few small incisions and takes about an hour. Recovery and rehabilitation take a few weeks. The procedure can reduce pain, improve mobility and stability, and get you back to life's activities. (Source: academic)	Timeline of recovery: What is the recovery time for meniscus repair surgery? Recovery can take 6-9 months, with a gradual return to activity. (Source: academic)
Evaluation of surgery: What is the success rate of arthroscopic meniscus repair? In general, meniscus repair surgery has a high success rate, from around 84% to 94%. (Source: medical practice)	Risks and complications: What are the risks of meniscus repair surgery? Risks include infection, nerve or blood vessel damage, and blood clots. (Source: academic)
Evaluation of surgery: Is it better to have a meniscus removed or repaired? Meniscal repair is a more biologically preserving procedure of the knee joint and should be performed preferentially over meniscectomy in isolated meniscal tears when feasible. (Source: government)	Evaluation of surgery: How successful is meniscus repair surgery? Success depends on tear location and type, with peripheral tears having higher success rates. (Source: academic)
Timeline of recovery: How long is bed rest after meniscus surgery? Complicated arthroscopic repair of a meniscus tear requires that the patient's knee be completely immobilized for 2 weeks after surgery. Followed by two weeks of limited motion before resuming daily activities. (Source: medical practice)	Technical details: What should I expect during meniscus repair surgery? The procedure involves anesthesia, small incisions, and the use of an arthroscope. (Source: academic)
Risks and complications: Will my knee ever be the same after meniscus surgery? Following meniscus surgery, a person may develop new knee pain. This can occur for several reasons, including reinjury or problems with postsurgical rehabilitation. It may also develop due to postsurgical complications, such as arthritis. The treatment for new knee pain may vary depending on the underlying cause. (Source: commercial)	Pain: Is meniscus repair surgery painful? Pain levels vary, but patients receive pain management and physical therapy post-surgery. (Source: academic)
Pain: Why does it hurt so much after arthroscopic knee surgery? After knee arthroscopy, you may experience spontaneous osteonecrosis. Also known as avascular necrosis, osteonecrosis is a painful disorder occurring when a bone doesn't receive sufficient blood supply. It causes pain and degradation of bone tissue. (Source: medical practice)	Timeline of recovery: How long will I be on crutches after meniscus repair? Typically, patients use crutches for 2-4 weeks. (Source: academic)​​​​​​​
Evaluation of surgery: Why is arthroscopy not recommended? And while complications from arthroscopy are rare, they can be serious, including blood clots and infections. There is also a relatively long recovery time (two to six weeks) and limited mobility after surgery. (Source: commercial)	Timeline of recovery: How soon can I return to sports after meniscus repair? Return to sports is generally advised after 6-9 months. (Source: academic)
Pain: What are the most painful days after meniscus surgery? You will be sent home from the surgery center with prescriptions for pain medication. Take the pain medication as prescribed. You take the medication and taper off as you feel comfortable, but keep in mind that many people have an increase in pain around day 3 or 4 after surgery. (Source: medical practice)	Evaluation of surgery: What are the benefits of arthroscopic surgery? Benefits include less pain, faster recovery, and smaller scars compared to open surgery. (Source: academic)
Special activities: How do you sit on the toilet after meniscus surgery? Youtube video. (Source: social media)	Technical details: Can meniscus repair be done on an outpatient basis? Yes, most surgeries are outpatient procedures. (Source: academic)
Restrictions: Do you need a knee brace after meniscus surgery? In most meniscus repair cases, the brace is recommended for the first six weeks after surgery. (Source: medical practice)	Longevity: What are the long-term problems after meniscus surgery? Potential issues include arthritis and spontaneous osteonecrosis. (Source: medical practice)
Special activities: Can I sleep sideways after meniscus surgery? Sleeping on your back can be an adjustment in and of itself. If you do choose to sleep on your side, roll to the non-surgery side and put a pillow between your knees. Use this position only if you're having no luck getting to sleep on your back, and remember not to bend the knee. (Source: commercial)	Technical details: Can meniscus repair be combined with other knee surgeries? Yes, it can be combined with procedures like anterior cruciate ligament (ACL) reconstruction. (Source: academic)
Restrictions: Do I sleep with a brace in after meniscus surgery? Knee immobilizer: meniscal repair patients are to wear the knee immobilizer full-time for the first three weeks to protect the repair for the first phase of healing. This includes while you are sleeping. It is to be removed only for physical therapy-directed exercises and showers. (Source: medical practice)	Timeline of recovery: How does meniscus repair compare to meniscectomy in terms of recovery? Meniscus repair typically requires a longer recovery time than meniscectomy. (Source: academic)

The recorded questions were categorized into subtopics according to the previously reported Rothwell system in the literature [14] (Table 2). Categorization of FAQs was performed by two independent reviewers in the event of discrepancy resolved by a third reviewer.

**Table 2:** Rothwell’s classification system for questions and websites Source: [14]

Rothwell's classification	Description
Fact	Asks whether something is true and to what extent, objective information
Policy	Asks whether a specific course of action should be taken to solve a problem
Value	Asks for evaluation of an idea, object, or event
Question classification by topic	Description
Fact	
Specific activities/restrictions	Ability/inability to perform a specific activity after surgery
Cost	Cost of surgery including questions about insurance coverage
Timeline of recovery	Specific questions regarding recovery and timelines
Technical details	The surgical procedure includes specific questions about surgery and anesthesia
Policy	
Indications	Surgical indications, alternatives, timing of surgery
Risks/complications	Risks/complications during and after surgery
Value	
Pain	Includes duration, severity, and management of pain
Evaluation of surgery	Evaluation of surgery, successfulness, or invasiveness
Website categorization	Description
Commercial	A commercial public organization that provides a source of health information
Academic	Institutions including universities, academic medical centers, academic societies, and journals
Medical practice	Local hospital or medical practice without an academic affiliation
Single surgeon	Websites of an individual surgeon
Government	Websites maintained by a national government organization

Statistical analysis

Statistical analyses were performed using Microsoft Excel (Microsoft, Redmond, USA). Cohen's kappa coefficients were calculated for interobserver reliability. The κ-value shows agreement among observers. Landis and Koch previously categorized κ-values of 0.00 to 0.20 as slight agreement; 0.21 to 0.40 - fair agreement; 0.41 to 0.60 - moderate agreement; 0.61 to 0.80 - substantial agreement; and 0.81 or greater refers to almost perfect agreement [15]. The kappa value for the interobserver reliability was 0.90 (excellent agreement) for website classification. For questions analysis, Fisher's exact test for proportions was performed to compare question categories and website classifications. Statistical significance was defined as a p-value less than 0.05.

## Results

A total of 30 questions, 15 from Google and 15 from ChatGPT, were recorded. Rothwell's classification system was used for each of the 15 questions.

**Figure 1:**
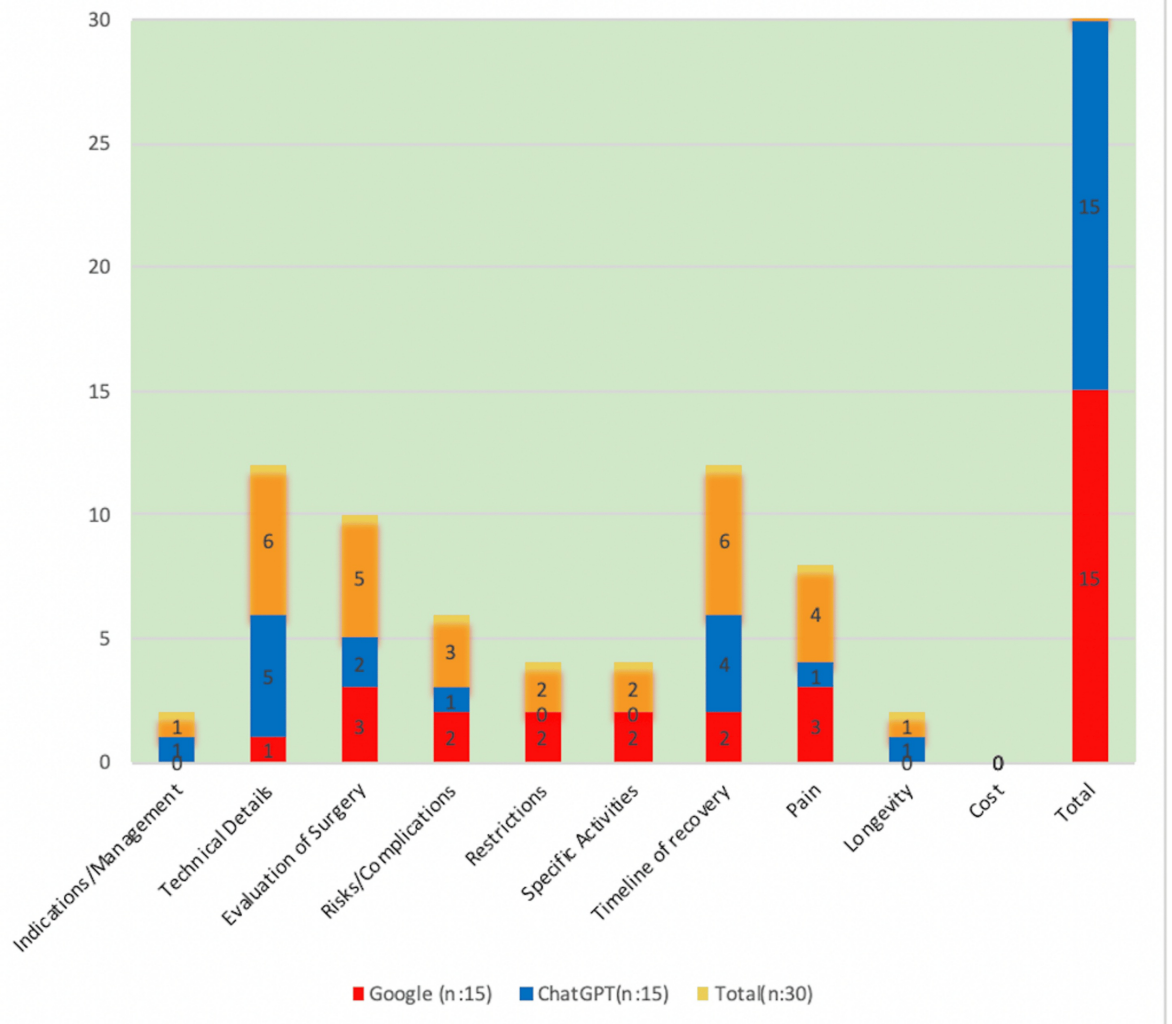
Categories of questions of ChatGPT and Google

Timeline of recovery (20%) and technical details (20%) were the most commonly asked question categories from a total of 30 questions. Technical details and timeline of recovery questions were more commonly asked on ChatGPT compared to Google (technical detail: 33.3% vs. 6.6%, p=0.168; timeline of recovery: 26.6% vs. 13.3%, p=0.651). The most commonly asked questions were in the technical details category (33.3%), followed by the timeline of recovery category (26%) in ChatGPT. The most commonly asked question categories in Google were evaluation of surgery (20%) and pain (20%). Categories of questions of ChatGPT and Google are presented in Figure 1. 

There were no questions in the cost category in both Google and ChatGPT. Google did not have any questions about indications/management and longevity categories. ChatGPT did not have any questions about either restrictions or specific activity categories. Percentage of questions by categories in Google and ChatGPT: indications/management - 0% vs. 6.6%, technical details - 6.6% vs. 33.3%, evaluation of surgery - 20% vs. 13%, risks/complications - 13.3% vs. 6.6%, restrictions - 13.3% vs. 6.6%, specific activities 13.3% vs. 0%, timeline of recovery - 13.3% vs. 26.6%, pain - 20% vs. 6.6%, longevity - 0% vs. 6.6%, cost 0% for both (see Table 3).

**Table 3:** Subcategories of most frequently asked questions

Classification/category	Google (n=15)	ChatGPT (n=15)	Total (n=30)	p-value
Question classification				
Indications/management	0	1	1	1.0000
Technical details	1	5	6	0.1686
Evaluation of surgery	3	2	5	1.0000
Risks/complications	2	1	3	1.0000
Restrictions	2	0	2	0.4828
Specific activities	2	0	2	0.4828
Timeline of recovery	2	4	6	0.6513
Pain	3	1	4	0.5977
Longevity	0	1	1	1.0000
Cost	0	0	0	-
Website categories				
Academic	3	14	17	0.0001
Medical practice	6	1	7	0.0801
Single surgeon	0	0	0	-
Government	2	0	2	0.4828
Commercial	3	0	3	0.2241
Social media	1	0	1	1.0000

Answers to questions were more commonly from academic websites in ChatGPT compared to Google (93.3% vs. 20%, p=0.0001). For ChatGPT, 14 out of 15 questions were answered by academic website references, of which four out of 15 (26.6%) were from the Hospital for Special Surgery, followed by Cleveland Clinic, University of Washington (UW) Orthopaedics, New York University (NYU) Langone Health, and Mayo Clinic which all of these sites were referenced in two out of 15 questions each (13.3%). ChatGPT had only one answer in the medical practice website category, which was about longevity. For a Google website search, most of the answers to FAQs were academic (20%) and commercial (20%).

**Figure 2:**
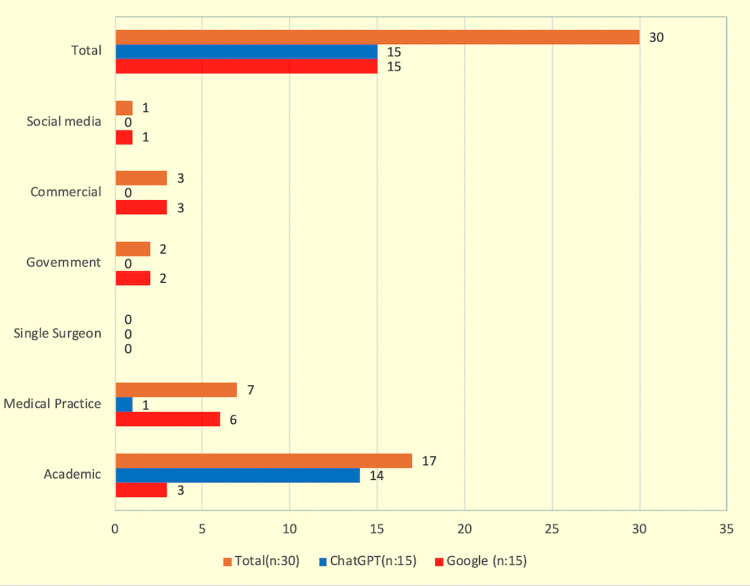
Types of website categories by source of ChatGPT and Google

## Discussion

The main finding of this study was that both ChatGPT and Google provide helpful information to frequently asked questions; however, ChatGPT provides answers from higher quality and more academic resources to FAQs about arthroscopic meniscus repair. One of the findings was that both ChatGPT and Google provided answers to frequently asked questions from different sources. When we further evaluated the sources, we found that ChatGPT provided answers from academic sources for almost all (14 out of 15) of the questions, while Google answered far less from academic references (93.3% vs. 20%, p=0.0001). Compared to Google, frequently asked questions on ChatGPT were more about technical details (33.3% vs. 6.6%) and timeline of recovery (26.7% vs. 13.3%). Compared to ChatGPT, Google provided answers mainly from medical practice websites (40% vs. 6.6%), and also frequently asked questions on Google were about the evaluation of surgery and pain (20% vs. 13% and 20% vs. 6.6%, respectively).

Due to rapid improvements in the field of AI, publications about the use and effects of AI are increasing in the literature [8,9,16]. Natural language processing models, such as ChatGPT, help patients retrieve information about their condition [17]. In our study, we aimed to analyze frequently asked questions on Google and ChatGPT and also detect the website sources of quoted answers. In this study, answers from ChatGPT were mostly from academic websites compared to Google (93.3% vs. 20%, p=0.0001), and this difference was statistically significant. Almost all of the answers (14 out of 15) were provided from academic websites in ChatGPT. Studies in the current literature show that ChatGPT provides comprehensive responses and uses more academic websites as references to FAQs [10,18-20]. Along with these recent publications about ChatGPT and Google comparison, we also found that ChatGPT mainly addresses academic websites, which may be interpreted as ChatGPT being a more objective and comprehensive tool compared to Google when the topic of arthroscopic meniscus surgery is concerned. ChatGPT commonly addresses academic websites of well-known organizations such as the Hospital for Special Surgery, Cleveland Clinic, and Mayo Clinic [20]. However, Google provided more references from medical practices (40% vs. 6.6%). Another finding of this study was that both Google and ChatGPT provide accurate information to frequently asked questions, but it can be seen that the source of answers is different as ChatGPT provides references from academic websites.

When current literature is reviewed for online questions and quality of online sources in various orthopedic fields such as spine, shoulder, hip, and knee arthroplasty, shoulder has shown that more common question categories were about technical details, indications/management, specific activities, and timeline of recovery [10,21-23]. In our study, the most commonly asked question categories overall were in technical details (six out of 30) and timeline of recovery (six out of 30). This may be the reason why academic websites are given for most of the references. One of the findings in the study is that the most commonly asked questions on Google were about pain (three out of 15) and evaluation of surgery (three out of 15). When we checked the question categories asked on ChatGPT, we can state that questions about technical details (five out of 15) and timeline of recovery (four out of 15) were the most commonly asked categories. These results show that FAQs in Google were more equally distributed, while on ChatGPT, some topics were more popular; however, in both ChatGPT and Google there were no FAQs in three subcategories. As can be seen in the literature, patients have inadequate knowledge about postoperative pain and its management [24]. Our analysis demonstrates that patients who want to obtain information about arthroscopic meniscus surgery need more information on topics such as technical details, timeline of recovery, evaluation of surgery, and pain.

By performing the study, we were able to compare ChatGPT and Google FAQs subcategories, categories of answer references, and sources, which allowed us to determine the quality of these tools. With this study, we also understand commonly asked questions about arthroscopic meniscus surgery by patients. There are multiple studies in the literature questioning the satisfaction and readability of the answers by patients for answers provided by ChatGPT and Google [25-28]. We can mention that ChatGPT's answers originate from mostly academic sources, providing ChatGPT with a more academic source in terms of answering frequently asked questions about arthroscopic meniscus repair.

There were some limitations in this study. Firstly, Google and ChatGPT are evolving, which may differentiate the source and accuracy of the answers provided. The rapidly evolving nature of AI models and search engines may limit the long-term applicability of these findings, necessitating periodic re-evaluation. Another limitation was although we believe that answers from academic sites are trustworthy, we cannot address whether this information is up-to-date since medical information is progressing rapidly.

## Conclusions

Compared to Google, ChatGPT provides significantly more academic references to questions about arthroscopic meniscus repair. However, it is crucial to acknowledge that while ChatGPT can assist in providing general information, it should not replace professional medical advice or personalized care. ChatGPT may be a valuable adjunct in patient education when used under physician supervision, ensuring that information aligns with evidence-based practices.

## References

[1] Kahan JB, Burroughs P, Petit L (2023). Rates of subsequent surgeries after meniscus repair with and without concurrent anterior cruciate ligament reconstruction. PLoS One.

[2] Luvsannyam E, Jain MS, Leitao AR, Maikawa N, Leitao AE (2022). Meniscus tear: pathology, incidence, and management. Cureus.

[3] Treuting R (2000). Minimally invasive orthopedic surgery: arthroscopy. Ochsner J.

[4] Friberger Pajalic K, Turkiewicz A, Englund M (2018). Update on the risks of complications after knee arthroscopy. BMC Musculoskelet Disord.

[5] Davenport T, Kalakota R (2019). The potential for artificial intelligence in healthcare. Future Healthc J.

[6] Bohr A, Memarzadeh K (2020). The rise of artificial intelligence in healthcare applications. Artif Intell Health.

[7] Kothari M, Moolani S (2015). Reliability of "Google" for obtaining medical information. Indian J Ophthalmol.

[8] Roblot V, Giret Y, Bou Antoun M (2019). Artificial intelligence to diagnose meniscus tears on MRI. Diagn Interv Imaging.

[9] Kim DH, MacKinnon T (2018). Artificial intelligence in fracture detection: transfer learning from deep convolutional neural networks. Clin Radiol.

[10] Tharakan S, Klein B, Bartlett L, Atlas A, Parada SA, Cohn RM (2024). Do ChatGPT and Google differ in answers to commonly asked patient questions regarding total shoulder and total elbow arthroplasty?. J Shoulder Elbow Surg.

[11] Kington RS, Arnesen S, Chou WS, Curry SJ, Lazer D, Villarruel AM (2021). Identifying credible sources of health information in social media: principles and attributes. NAM Perspect.

[12] Koenig S, Nadarajah V, Smuda MP, Meredith S, Packer JD, Henn RF 3rd (2018). Patients' use and perception of internet-based orthopaedic sports medicine resources. Orthop J Sports Med.

[13] McCormick JR, Kruchten MC, Mehta N (2023). Internet search analytics for shoulder arthroplasty: what questions are patients asking?. Clin Shoulder Elb.

[14] Khalil LS, Castle JP, Akioyamen NO, Corsi MP, Cominos ND, Dubé M, Lynch TS (2023). What are patients asking and reading online? An analysis of online patient searches for rotator cuff repair. J Shoulder Elbow Surg.

[15] Landis JR, Koch GG (1977). The measurement of observer agreement for categorical data. Biometrics.

[16] Bitkina OV, Park J, Kim HK (2023). Application of artificial intelligence in medical technologies: a systematic review of main trends. Digit Health.

[17] Giorgino R, Alessandri-Bonetti M, Del Re M, Verdoni F, Peretti GM, Mangiavini L (2024). Google Bard and ChatGPT in orthopedics: which is the better doctor in sports medicine and pediatric orthopedics? The role of ai in patient education. Diagnostics.

[18] Chen Y, Zhang S, Tang N, George DM, Huang T, Tang J (2024). Using Google web search to analyze and evaluate the application of ChatGPT in femoroacetabular impingement syndrome. Front Public Health.

[19] Shen OY, Pratap JS, Li X, Chen NC, Bhashyam AR (2024). How does ChatGPT use source information compared with Google? A text network analysis of online health information. Clin Orthop Relat Res.

[20] Oeding JF, Lu AZ, Mazzucco M (2024). ChatGPT-4 performs clinical information retrieval tasks using consistently more trustworthy resources than does Google search for queries concerning the latarjet procedure. Arthroscopy.

[21] Mastrokostas PG, Mastrokostas LE, Emara AK (2024). GPT-4 as a source of patient information for anterior cervical discectomy and fusion: a comparative analysis against Google web search. Global Spine J.

[22] Obana KK, Lind DR, Mastroianni MA, Rondon AJ, Alexander FJ, Levine WN, Ahmad CS (2024). What are our patients asking Google about acromioclavicular joint injuries?-frequently asked online questions and the quality of online resources. JSES Rev Rep Tech.

[23] Shen TS, Driscoll DA, Islam W, Bovonratwet P, Haas SB, Su EP (2021). Modern internet search analytics and total joint arthroplasty: what are patients asking and reading online?. J Arthroplasty.

[24] Nasir M, Ahmed A (2020). Knowledge about postoperative pain and its management in surgical patients. Cureus.

[25] Johns WL, Martinazzi BJ, Miltenberg B, Nam HH, Hammoud S (2024). ChatGPT provides unsatisfactory responses to frequently asked questions regarding anterior cruciate ligament reconstruction. Arthroscopy.

[26] Li LT, Sinkler MA, Adelstein JM, Voos JE, Calcei JG (2024). ChatGPT responses to common questions about anterior cruciate ligament reconstruction are frequently satisfactory. Arthroscopy.

[27] Johns WL, Kellish A, Farronato D, Ciccotti MG, Hammoud S (2024). ChatGPT can offer satisfactory responses to common patient questions regarding elbow ulnar collateral ligament reconstruction. Arthrosc Sports Med Rehabil.

[28] Fahy S, Oehme S, Milinkovic D, Jung T, Bartek B (2024). Assessment of quality and readability of information provided by ChatGPT in relation to anterior cruciate ligament injury. J Pers Med.

